# 

**Published:** 1860-09

**Authors:** 


### CONTENTS.

### ORIGINAL COMMUNICATIONS.

PAGE.

ARTICLE I.—Spider Bite.—Severe Symptoms and Unusual Phenomena—Recovery. By J. T. Banks, M. D., Griffin, Ga.,....... 2

ARTICLE II.—Synopsis of the Clinic Lectures of Prof. John W. Jones, in the Atlanta Medical College. Session 1860,........... 5

ARTICLE III.—Medical Clinic for the Session of 1860. Service of Dr. J. G. Westmoreland,.................................... 10

ARTICLE IV.—Ethereal Tinct. Valerian in Convulsions in Children. By B. W. Hardee, M. D., Savannah, Ga.,......................... 13

### SELECTIONS.

The Conditions attending every Attack of Acute Rheumatism. By Albert T. Wheelock, A. M., M. D., author of Boylston Prize Essay for 1838. Belfast, Maine,.................................... 16

Caffeine in Opium-Coma.—The Second Case of the Injection of Caffeine, by the Rectum, in Extreme Narcotism of Opium. By Henry F. Campbell,................................................ 20

The Ophthalmoscope and its use in the Diagnosis of the Diseases of the deeper parts of the Eye. By J. W. Hulke, F. R. C. S., Assistant Surgeon to the Royal London Ophthalmic Hospital, Moorfields, and to King's College Hospital, London,................. 24

Puerperal Eclampsia, treated by Subcutaneous injections of Morphia. By Scanzoni, of Strasburg. (Bull. de Therap.)................. 31

Anæsthetics in Hospital Practice,.................................... 35

A Case of Purpura Urticans. By Wm. H. Doughty, M. D.,......... 39

On the Climate of California in its relation to the Treatment of Pulmonary Consumption. By James Blake, M. D., F. R. C. S.,....... 41

On the Treatment of Typhoid Forms of Scarlatina by Tonics, Alcoholic Stimulants and Food. By Benjamin Cutter, M. D., of Woburn,..................................................... 47

### EDITORIAL AND MISCELLANEOUS.

Valedictory,........................................................ 51

Salutatory,......................................................... 52

Linseed Oil in Consumption,........................................ 56

Larch Bark in Hæmoptysis,.......................................... 55

Calomel in Sickness of Pregnancy,................................... 56

Nourishment in Typhoid Fever,...................................... 57

American Medical Association,...................................... 59

Insanity in Paris,................................................... 63

Payments,........................................................... 63

Meteorological Table,............................................... 64

